# Age density patterns in patients medical conditions: A clustering approach

**DOI:** 10.1371/journal.pcbi.1006115

**Published:** 2018-06-26

**Authors:** Fahad Alhasoun, Faisal Aleissa, May Alhazzani, Luis G. Moyano, Claudio Pinhanez, Marta C. González

**Affiliations:** 1 Center for Computational Engineering, Massachusetts Institute of Technology, Cambridge, Massachusetts, United States of America; 2 Center for Complex Engineering Systems at KACST and MIT, Cambridge, Massachusetts, United States of America; 3 Media Lab, Massachusetts Institute of Technology, Cambridge, Massachusetts, United States of America; 4 IBM Research Labs, Rio de Janeiro, Brazil; 5 Department of City and Regional Planning, University of California, Berkeley, Berkeley, California, United States of America; 6 Lawrence Berkeley National Laboratory, Berkeley, California, United States of America; University of Calgary Cumming School of Medicine, CANADA

## Abstract

This paper presents a data analysis framework to uncover relationships between health conditions, age and sex for a large population of patients. We study a massive heterogeneous sample of 1.7 million patients in Brazil, containing 47 million of health records with detailed medical conditions for visits to medical facilities for a period of 17 months. The findings suggest that medical conditions can be grouped into clusters that share very distinctive densities in the ages of the patients. For each cluster, we further present the ICD-10 chapters within it. Finally, we relate the findings to comorbidity networks, uncovering the relation of the discovered clusters of age densities to comorbidity networks literature.

## Author summary

Age and sex of a patient can be directly related to susceptibilities to certain medical conditions. We present a method to generate clusters of human phenotype, based on the age of the population. This method helps extract knowledge on age and sex from the data. The age and sex correlations with disease conditions can help in a task of predicting the susceptibility of incoming patients to conditions.

## Introduction

Studies of groups of diseases occurring together, or disease comorbidities, have traditionally focused on studies of small groups of diseases using techniques of hypothesis-testing [1–6]. The repeated existence of particular comorbidities is important to diagnoses and better index diseases [7, 8]. Databases of electronic medical records contain phenotypic information for humans—namely, patient clinical histories. A novel method to analyze health records is to built the human phenotypic disease network, where nodes represent the diseases and edges indicate comorbidity relations [9]. More recent studies analyze databases on electronic health records to uncover systematic associations in the complete set of known diseases [6, 10–12]. In this context, several methods of information sciences can be used to uncover patterns in electronic patient records. The main interest of these studies is to discover correlations between diseases that can help in prevention and can also inform systems biology frameworks [13]. More recently, computational methods are being used to reduce the costs of healthcare by helping to identify outliers in medical records [14].

Up to date, most of the samples of electronic patient records studied in the literature have used a narrow set of the general population of patients. For example, Hidalgo *et al*. covered 3 years of medical care claims of patients who were 65 years or older, this biased the information towards population of the elderly. Later, Roque *et al*. generated fine grained patient stratification and disease co-occurrence statistics of patients from the Sankt Hans Hospital, which is the largest Danish psychiatric facility [15]. Their results focus into phenotypes associated with mental and behavioral disorders or the chapter V of the ICD-10 standard classification catalog. Datasets with more complete sample of the population have become more recently available. Electronic records with time spans of decades allowed, for the first time, to uncover patterns centered on the number of key diagnoses that can detect diseases earlier in a patient’s life [16]. While Chmiel *et al*. [17] analyzed two years of medical claims of the entire population in Austria. They measured how the comorbidity network change its structure with the age of the patients. This information was used to build a diffusion model that explains a large percentage of the variance of all the disease incidents in a population. In that case, the comorbidity networks were built while pre-defining the age intervals of the patients analyzed. In this work, we present a clustering method by identifying the similarities in the age densities of the actual phenotypic records. We find groups of medical conditions that occur in the unsupervised age groups emerging from the data. These groups are in turn associated with a small set of chapters of the ICD-10 standard classification catalog.

The wisdom of doctors when it comes to assessing susceptibility to diseases have been influenced by the years of practice and observation of many cases on daily basis. Doctors’ knowledge of the susceptibility to diseases at different ages/sexes serves as an essential prior to perform diagnostics of incoming patients. Similar symptoms for patients might lead to different diagnosis depending on the age and sex of the patient, a patient who is 70 years old is much more likely to suffer a heart attack than a 10 year-old even if both patients are suffering the symptom of chest pain. We show here that this common knowledge can be inferred from the data. Besides the symptoms a patient is having, the age and sex can aid the diagnostic process. We present a framework that automatically uncovers the relationship between health conditions and the age/sex of a patient. To that end, we group the health conditions based on their similarities in population age densities. Then, we construct the comorbidity graph in the same way found in the literature [9, 17] to investigate the relationship of comorbidity coefficient values to the discovered clusters of conditions.

## Results

For each of the 1.7 million patients there is a log for each visit to the doctor within the 17 months of study from March, 2013 to July, 2014. The data corresponds to medical claims from one of the largest healthcare insurance companies in Brazil. Each health record in the database has several attributes pertaining to the data of the visit, it is synthesized via ICD-10 codes that detail the condition and the purpose of the visit. ICD-10 codes have a range of 23 thousands of different identifiers each representing a health condition of a patient. In addition, the data has the age and sex of each patient. The total number of visits is 6.6 millions, resulting in 47 million conditions.

**Fig 1:**
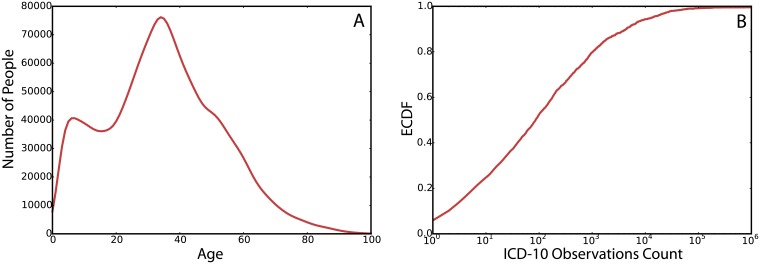
(A) The distribution of age in patients. (B) The cumulative density function of the ICD-10 codes by the number of observations in the data.

In Fig 1A we show the age distribution of the entire population of patients in the data. With the age distribution peaking at 34 which is the median age, in agreement with the median age of the entire population of Brazil.

Fig 1B shows the cumulative distribution of the frequencies of ICD-10 codes in the data. About 50% of the ICD-10 codes had a frequency of less than 100 times among all patients’ visits, while the rest of the 50% of the codes makes up for 90% of the records in the data set.

**Fig 2:**
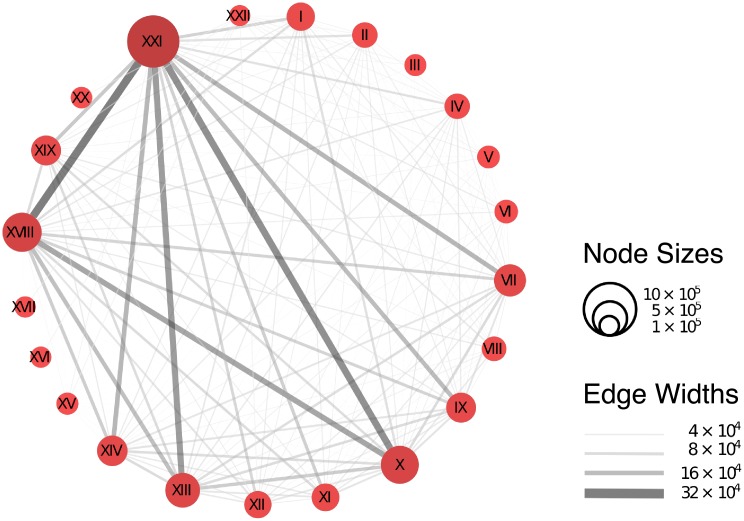
Network representation of the ICD-10 grouped in their 22 category chapters. The weight of the links represent the number of co-occurrences in the patients records and the size of the nodes represent the frequency of each chapter.

The ICD-10 coding scheme is structured in a tree and the top level contains 22 chapters. The chapters of ICD-10 have common characteristics pertaining to the same organ/system or relating to the nature of the visit. The size of the chapter nodes in Fig 2 corresponds to the frequency of observing the chapter in the data. The thickness of the edges between the nodes corresponds to the frequency of co-occurrence of the chapters in the patients records. Chapter XXI has the highest frequency in the data, it is described as factors influencing health status and contacts with health services, such as performing routine checkups. Chapter X contains the group of conditions relating the respiratory system; VII are diseases of the eye and adnexa; XIII are diseases of the musculoskeletal system and connective tissue and XVIII are abnormal clinical and laboratory findings not elsewhere classified. The description of all the chapters of disease codes is included in (S1 Table).

**Fig 3:**
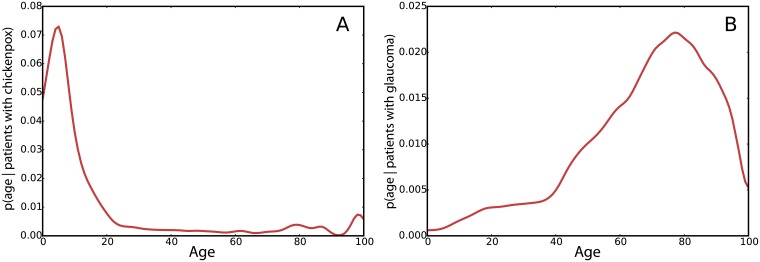
Age distribution for patients with Chickenpox (A) and Glaucoma (B).

By inspection, each ICD-10 code has a distinctive signature of density on the age dimension that spans the various age groups from birth onward. Fig 3 shows example age density signatures of Chickenpox and Glaucoma. As expected, Glaucoma is more prevalent for the older group [18] and Chickenpox in kids [19, 20]. The shapes of these distributions hint that there is a pattern of higher likelihood of patients of a certain age for different diseases.

### Clustering ICD-10 codes

We further analyze the age densities of ICD-10 codes in the data to segment ICD-10 codes into groups of conditions with similar age densities. As a robustness measure, we consider the analysis by excluding all codes of chapters XVIII-XXII. The excluded chapters include symptoms (e.g. R codes), procedural details (such as complications or adverse drug effects) and also personal factors (general examinations and such). We represent the age distribution as a vector of 100 elements, each element has the probability of a patient of the corresponding age within the population of patients having the code. This is defined as probability *p*(*age*|*patient* ∈ *c*) where *age* is the age of the patients, *c* is a disease code and *patient* ∈ *c* is the set of patients that had a visit labeled as *c*.

**Fig 4:**
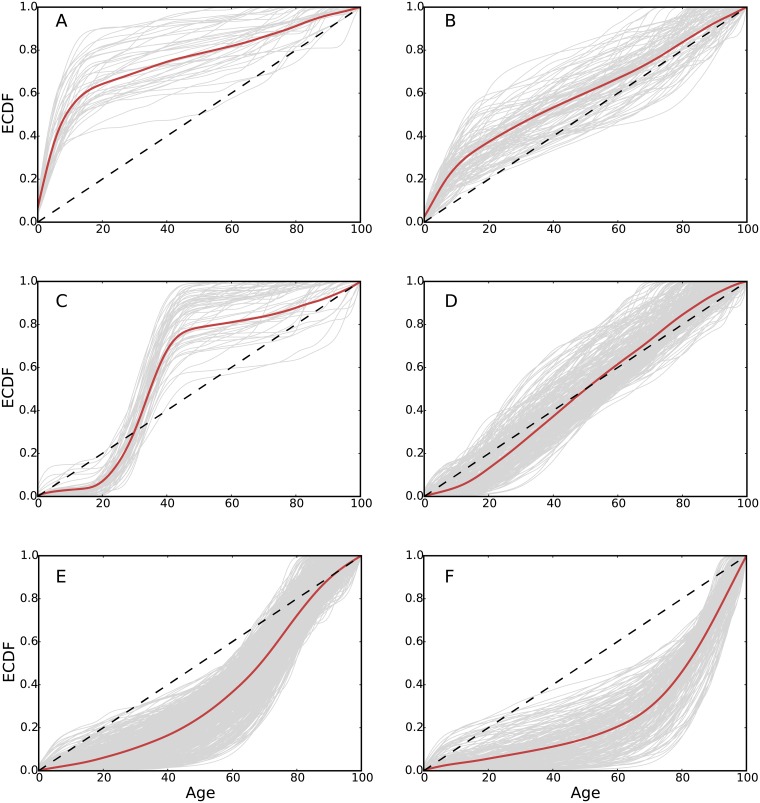
Lines in gray represent cumulative distribution of *P*(*age*|*patients* ∈ *c*) and lines in red are the cluster averages for illustration. The clusters of ICD-10 codes given by the HAC are labeled from A to F. Cluster A of ICD-10 codes have more concentration towards infants and children. Cluster B of diseases having a density closer to a uniform but with a tendency to have relatively more concentration in teenage years and early adulthood. Cluster C has the narrowest concentration of age in the thirties. Cluster D groups codes that distribute uniformly in all ages. Cluster E groups codes for ages over 60. Cluster F groups ICD-10 codes in patients over 70.

We cluster the densities for each ICD-10 code based on the vector representation of the age density *p*(*age*|*patient* ∈ *c*). We use Hierarchical Agglomerative Clustering (HAC) to group the codes into clusters. The method is further discussed in the material and methods section. The age distribution of the codes clusters into six main groups as shown in Fig 4. Clusters A and B show two clusters of codes having higher density towards the lower spectrum of ages. Cluster C shows a group of codes that have age densities concentrated in the ages 20 to 40. Cluster D has diseases that are almost uniformly distributed across the ages. Cluster E has codes with densities concentrating in the range of ages over 60 and cluster F has codes with age densities concentrating over 70. The kernel density estimation of the probability density of the clusters is included in (S1 Fig).

**Fig 5:**
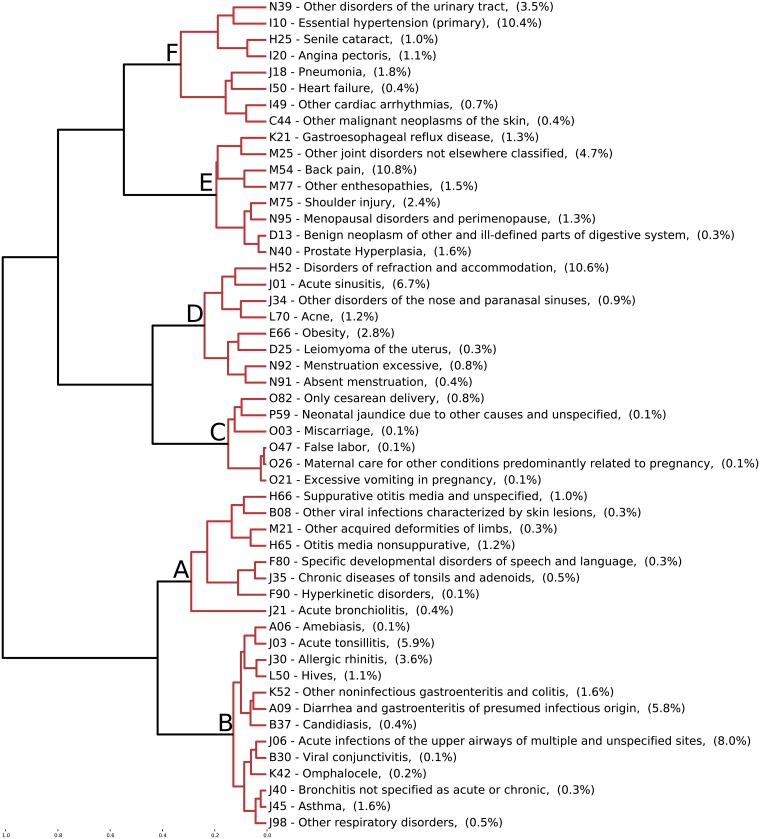
Hierarchical clustering with a depth of six in the dendrogram tree, branches of depth higher than six are represented by the ICD-10 code that is most common in that branch. The frequency of each ICD-10 code is in parenthesis in percentage of the total population of patients. The alphabet letters assignments correspond to the clusters discussed in Fig 4.

Fig 5 illustrates a few examples of the high prevalent ICD-10 codes from the clusters discovered in the data. For each cluster, Fig 5 shows the clustering dendrogram with a depth of six, branches in the dendrogram with a depth higher than six are represented by the disease that is most common in their respective branch. The branches are labeled by their clusters from A to F. Within cluster A, J21 acute bronchiolitis and H65 otitis media nonsuppurative were observed in 0.4 and 1.2 percents of the population respectively, both have a concentration towards the lower ages as shown previously. Cluster B has J06 acute infections of the upper airways with 8 percents of the population of patients. Furthermore, it has A09 diarrhea and J03 acute tonsillitis each with around 5.9 percents respectively. The noticeably increase of the percentage of patients is due to the population age distribution shown in Fig 1. Cluster C with O82 Cesarean delivery has around 0.8 percents of the population of patients, the cluster is consistent with the defined age range between 20 and 40. Cluster D has H52 disorders of refraction and accommodation with 10.6 percents and J01 acute sinusitis with 6.7 percents of the population of patients. As expected, as the clusters have more density around the peak of the age distribution of the population, the number of patients per code in the clusters becomes higher. Cluster E with age density towards the elderly has M54 back pain with 10.8 percents and M25 other joint disorders with 4.7 percents as the most common. Cluster F with age density in the oldest group has I10 essential hypertension (primary) with 10.4 percents and N39 other disorders of the urinary tract with around 3.5 percents. Pneumonia is third in around 1.8 percents.

**Fig 6:**
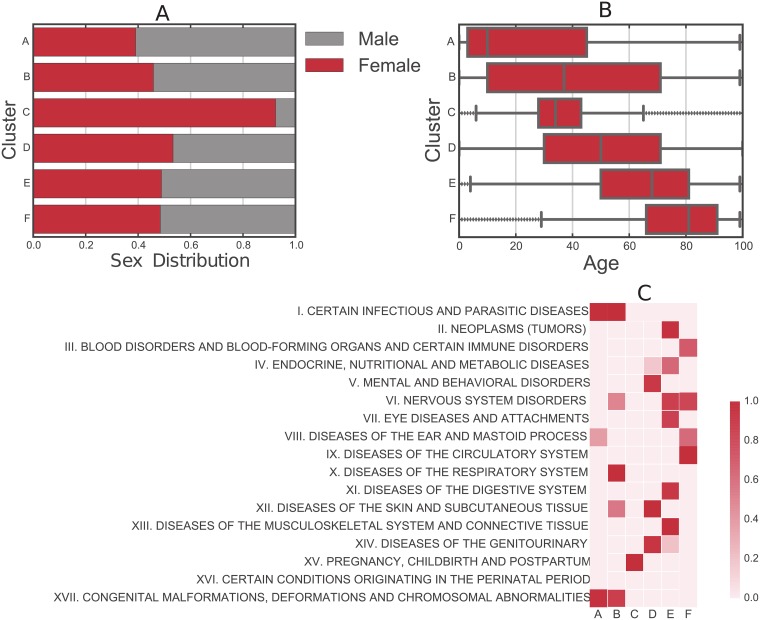
Patient characteristics per cluster. (A) Sex distribution. (B) Age distribution. (C) Probability of associations between our identified clusters and the category chapters of ICD10 codes (1 − (*p*- value)). The alphabet letters correspond to the clusters discussed in Fig 4.

Fig 6 shows the decomposition of the clusters in terms of sex and age distribution of each cluster, which has the expected results. Further, we show the probability of association between clusters and the ICD-10 chapters agreed by the World Health Organization [21], we use the Fisher exact test to measure the association between a chapter of codes to our identified clusters. Clusters have increasing mean age except for cluster C where the age range concentrated around 34. Cluster C is dominated by female patients. This is explained by the high probability of association with ICD-10 codes in chapter XV pertaining to pregnancy and childbirth and postpartum. Interestingly, from A to D each cluster has their own signature of few associated chapters, while E and F are associated with more chapters proper of aging.

### Comorbidity and clusters analysis

**Fig 7:**
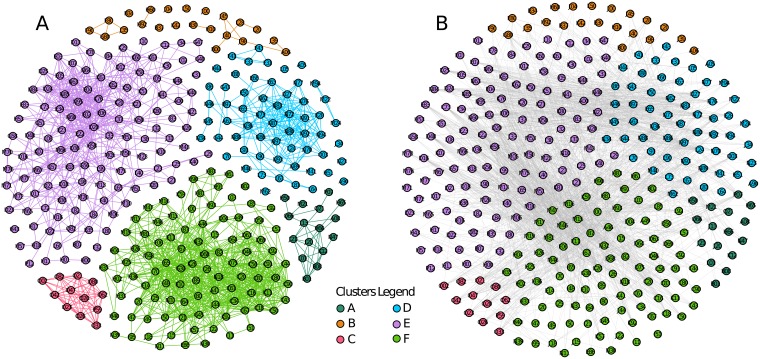
The comorbidity network of highest two thousand values of relative risk (i.e. comorbidity). Nodes in the network are ICD-10 codes and edges represent the relative risk between the disease codes, the edges displayed in the figure belong to the highest two thousand relative risk values for purposes of visualization. Edges in the network (A) show intra-cluster comorbidities and edges in network (B) shows the inter-cluster comorbidities.

This section sheds light on the age related characteristics of the edges in comorbidity networks [9, 17]. We first construct the comorbidity network through the measure of relative risk between conditions. Further details about the measure of relative risk are included in the materials and methods. Fig 7 shows a sample of the comorbidity network. In the figure, we only show the edges with highest two thousand relative risk values in the quantified comorbidities. The figure is splitted into two parts A and B. Part A shows the intra-cluster edges and part B shows the inter-cluster edges. The sample selection of edges and nodes display are done for visualization purposes.

**Fig 8:**
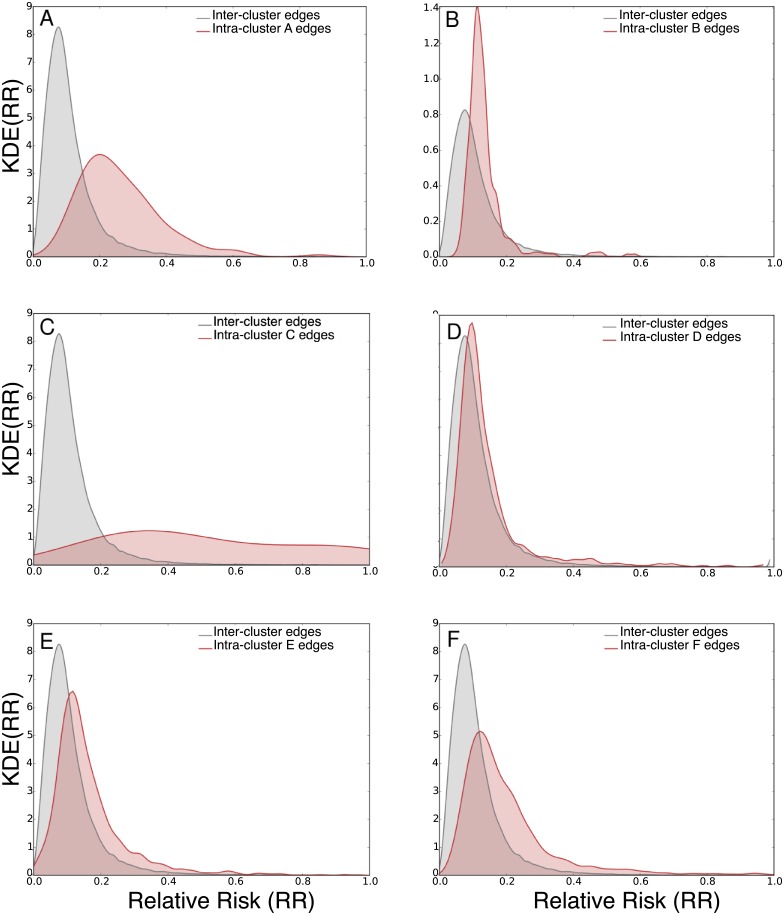
The distribution of relative risk for inter versus intra cluster edges. In gray is the distribution of relative risk of inter-cluster edges. In red are the distributions of relative risk for intra-cluster edges for the respective cluster.

To relate the clusters of diseases reported earlier to the study of comorbidity networks, we study the distribution of relative risk for inter-cluster versus intra-cluster comorbidities. Fig 8 shows the distributions of the relative risk of inter-cluster versus intra-cluster comorbidities. For each cluster in the data, we quantify the distribution of the relative risk of intra-cluster comorbidities (in red) and plot it against the distribution of the relative risk of inter-cluster comorbidities (in gray).

We find a clear variation in the divergence between the density of relative risk for inter-cluster comorbidities to the intra-cluster ones. The closer the distribution of age for patients in a cluster to a uniform distribution, the less the divergence in relative risk between inter and intra cluster edges. The divergence is highest in clusters A, C and F. They belong to the clusters that identify infants, women in reproductive age and the elderly. It is followed by clusters E and B. With E grouping age density towards the elderly with M54 back pain patients and M25 other joint disorders, while B groups conditions with concentration in teenage years and early adulthood. Cluster D is the closest in age distribution to a uniform, and has the minimal divergence to the distribution of inter-cluster comorbidity density, which has patients in H52 disorders of refraction and accommodation and J01 acute sinusitis.

## Discussion

This paper presents an approach towards investigating groups of diseases based on their relation to age and sex using the records of medical visits from a diverse population. We show that besides the symptoms, age and sex can rank the susceptibility to conditions in a diagnostic process. Using Hierarchical Agglomerative Clustering, we uncover 6 significant groups of medical conditions that present strong similarities on the age density of the patients. Each group of these medical conditions has meaningful associations with few of the 22 standard chapters used to categorize diseases. To find these associations we use the Fisher exact test. We relate the found groups of conditions to the study of comorbidity networks. Pairs of conditions tend to have higher relative risk with varying magnitudes when conditions are in the same group (intra-cluster conditions) compared to conditions that are not in the same group (inter-cluster conditions). This in a sense means that the correlations of conditions in terms of sex and gender partially explain the higher relative risk values discovered in comorbidity networks [9, 17]. Our findings build prior knowledge related to age and sex for automated diagnostics in a Bayesian setting to predict the condition of a patient given their symptoms. The code and data of the study are available for access at http://www.github.com/fha/brazil_health_study.

## Materials and methods

### Ethics statement

This paper studies a population of 1.7 million patients in Brazil, containing 47 million of health records with detailed medical conditions for visits to medical facilities for a period of 17 months. The data were analyzed anonymously for the privacy of patients’ data.

### Hierarchical agglomerative clustering of ICD-10 codes

To uncover common patterns of the age distribution of ICD-10 codes, we used a Hierarchical Agglomerative Clustering (HAC) approach to group the codes based on the similarities of age distributions. Each code is represented by a vector *v* of length 100 where each cell represents *p*(*age* = *i*|*patients* ∈ *c*) where *patients* ∈ *c* is the set of patients with the condition on their records.

HAC cluster vectors, where each vector is a representation of the probability mass function of a code in the data. The vector representation of the probability mass function of the ages of a ICD-10 code is as follows:

$$p\left(age|patient\in code\right)=\left[p_{1},p_{1},....,p_{100}\right]$$ (1)

Where *p*_*i*_ = *p*(*age* = *i*|*patient* ∈ *code*) for a given code. At initialization, HAC assigns each vector object to a cluster, and sequentially merging them into clusters until all codes form one cluster. For measuring the distance *d* between two vector representations of age density, we use euclidean distance. The Ward distance criterion of clusters is dependent on the within cluster distances and the across clusters distances. Ward algorithm is conservative when merging clusters, thus it tends to find very compact clusters [22].

HAC provides a hierarchy structure of the clustered codes as illustrated in Fig 4. To determine the number of clusters *k* that best divide the data, we calculate the total within-cluster distances for *k* from 1 to 20. The total of distances drops as *k* increases until it does not decrease significantly. We select *k* that corresponds to the point where the total distances stops decreasing significantly. This method is known as the elbow curve method.

### Relative risk and comorbidity

To quantify the comorbidity between conditions, we employ a similar measure to what is used in the literature [9, 17]. We used the relative risk measure to quantify the comorbidity between conditions in the dataset. The formula for quantifying the relative risk between two conditions is given by:

$$RR_{ij}=\frac{C_{ij}N}{P_{i}P_{j}}$$ (2)

Where *C*_*ij*_ is the number of patients having both *i* and *j* diseases, *N* is the total number of patients in the data. *P*_*i*_ is the prevalence of condition *i* and *P*_*j*_ is the prevalence of condition *j*.

## Supporting information

S1 Table Description of ICD-10 chapters. (DOCX)

S1 Fig Probability density function of clusters by age. Kernel density estimation for a sample of disease codes from each cluster. Lines in gray represent probability distribution of *P*(*age*|*patients* ∈ *c*) and lines in red are the cluster averages for illustration. The clusters of ICD-10 codes given by the HAC are labeled from A to F.
(PDF)
