# Supplementary material for: Age density patterns in patients medical conditions: A clustering approach
Source: PLoS Comput Biol. 2018 Jun 26;14(6):e1006115. doi: 10.1371/journal.pcbi.1006115 (PMC6037375; doi:10.1371/journal.pcbi.1006115)
Supplement: S1 Table — (DOCX) [file pcbi.1006115.s001.docx]

| Chapter I | Certain infectious and parasitic diseases |
| --- | --- |
| Chapter II | Neoplasms [tumors] |
| Chapter III | Blood disorders and blood-forming organs and certain immune disorders |
| Chapter IV | Endocrine, nutritional and metabolic diseases |
| Chapter V | Mental and behavioral disorders |
| Chapter VI | Nervous system disorders |
| Chapter VII | Diseases of the eye and annexes |
| Chapter VIII | Diseases of the ear and mastoid process |
| Chapter IX | Diseases of the circulatory system |
| Chapter X | Diseases of the respiratory system |
| Chapter XI | Diseases of the digestive system |
| Chapter XII | Diseases of the skin and subcutaneous tissue |
| Chapter XIII | Diseases of musculoskeletal system and connective tissue |
| Chapter XIV | Diseases of the genitourinary |
| Chapter XV | Pregnancy, childbirth and postpartum |
| Chapter XVI | Certain conditions originating in the perinatal period |
| Chapter XVII | Congenital malformations, deformations and chromosomal abnormalities |
| Chapter XVIII | Symptoms, signs and abnormal clinical and laboratory findings, not elsewhere classified |
| Chapter XIX | Injury, poisoning and certain other consequences of external causes |
| Chapter XX | External causes of morbidity and mortality |
| Chapter XXI | Factors influencing health status and contact with health services |
| Chapter XXII | Codes for special purposes |
