# Supplementary figures and images for: Age density patterns in patients medical conditions: A clustering approach
Source: PLoS Comput Biol. 2018 Jun 26;14(6):e1006115. doi: 10.1371/journal.pcbi.1006115 (PMC6037375; doi:10.1371/journal.pcbi.1006115)

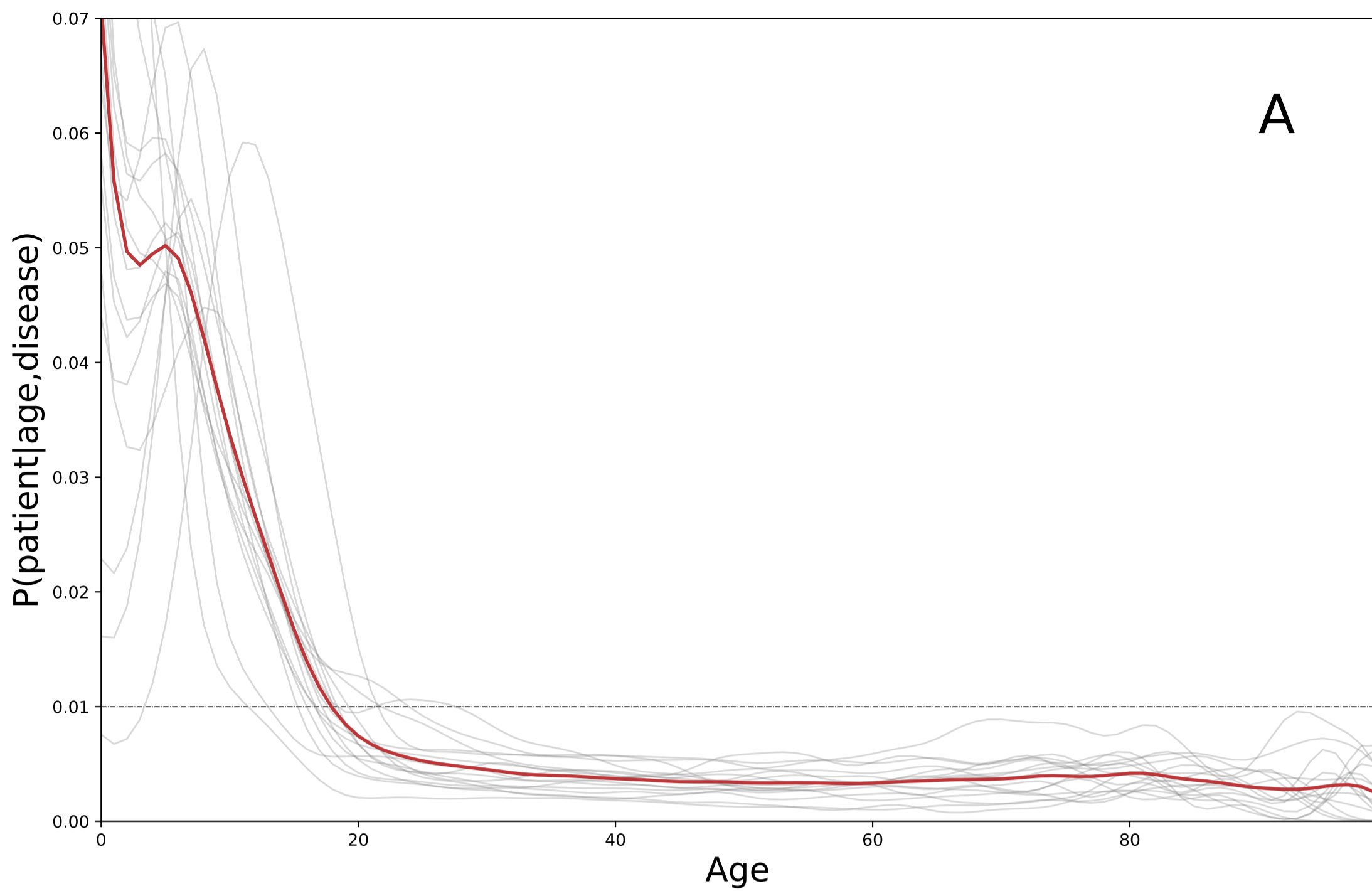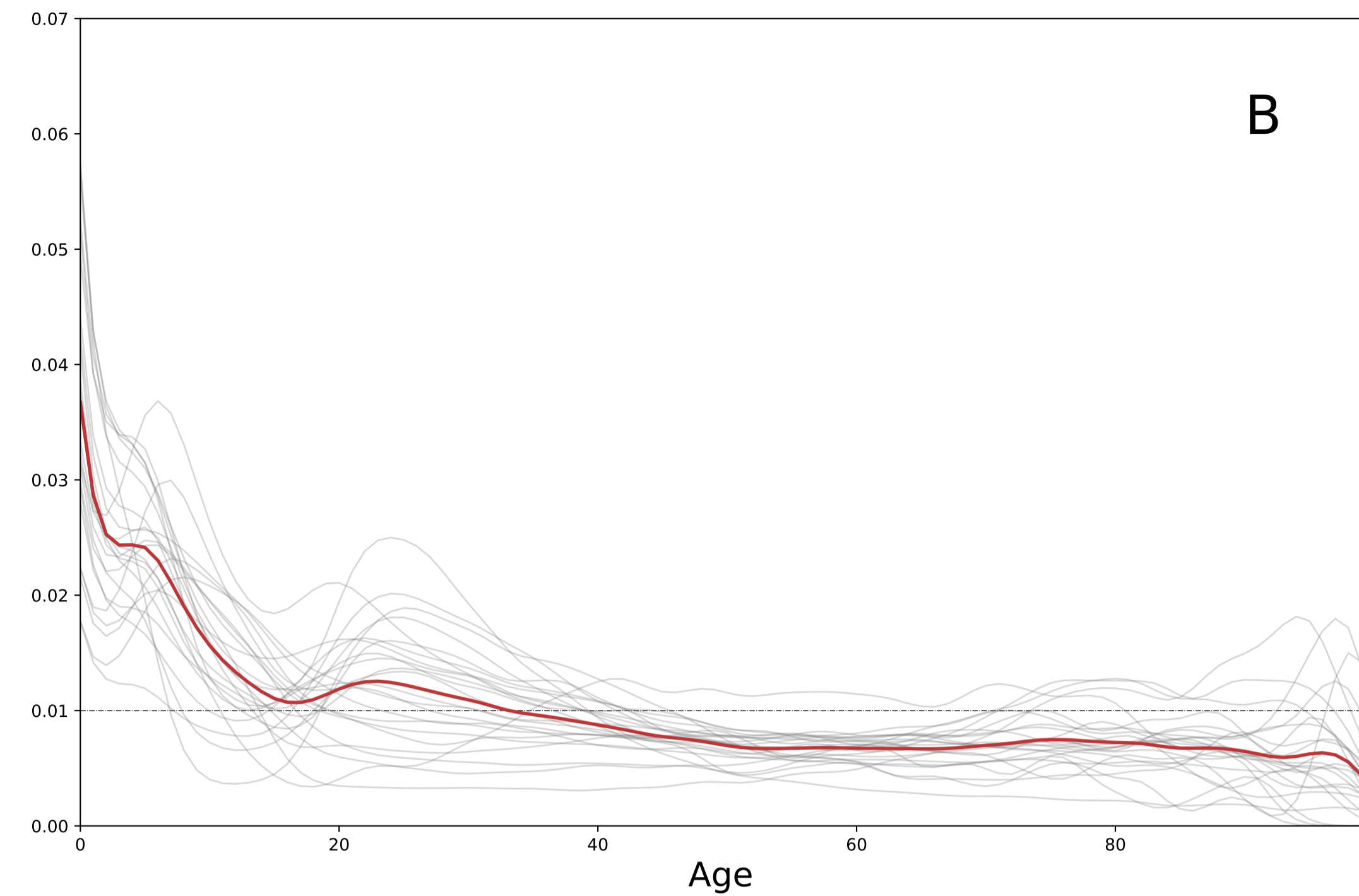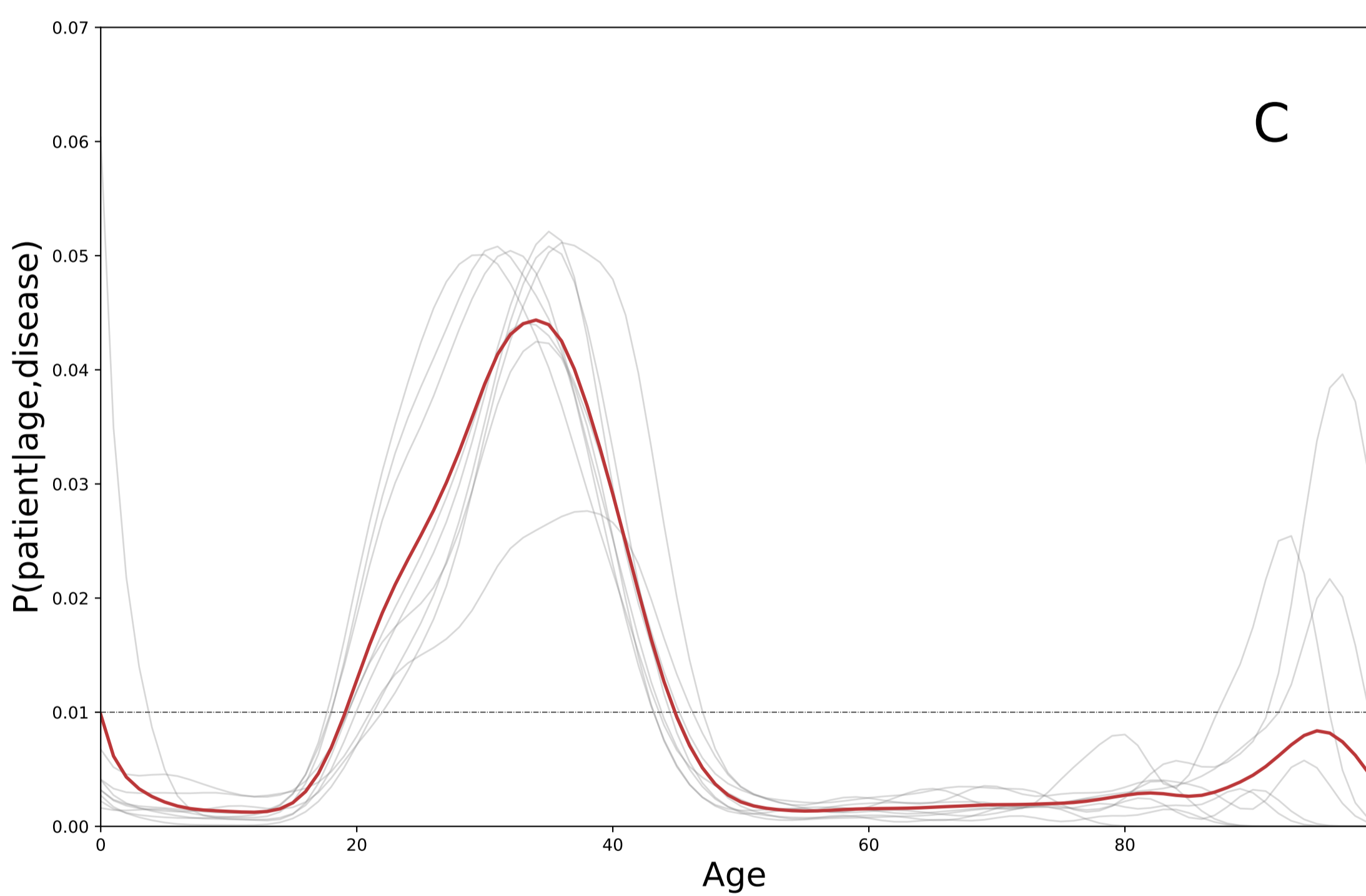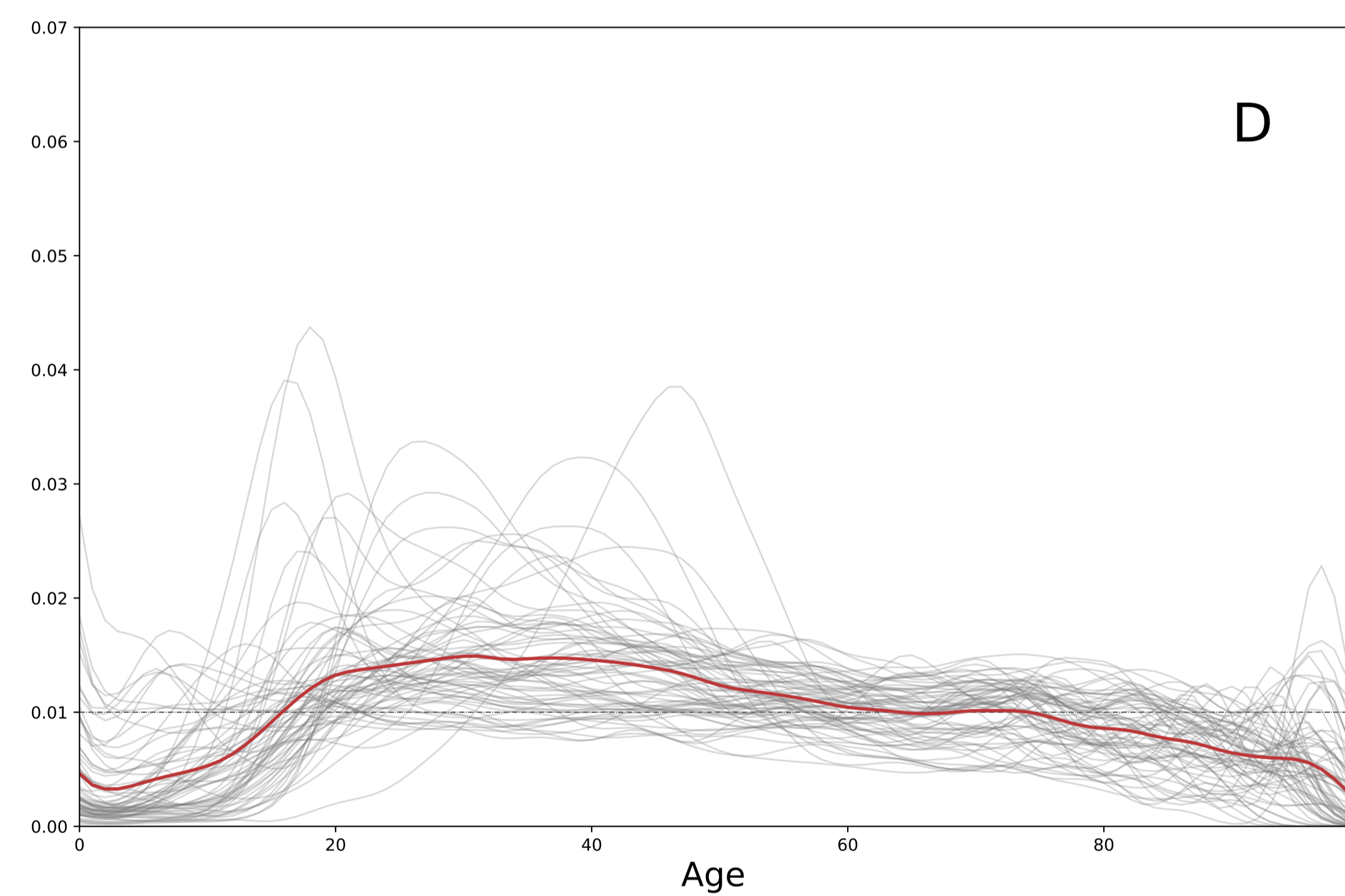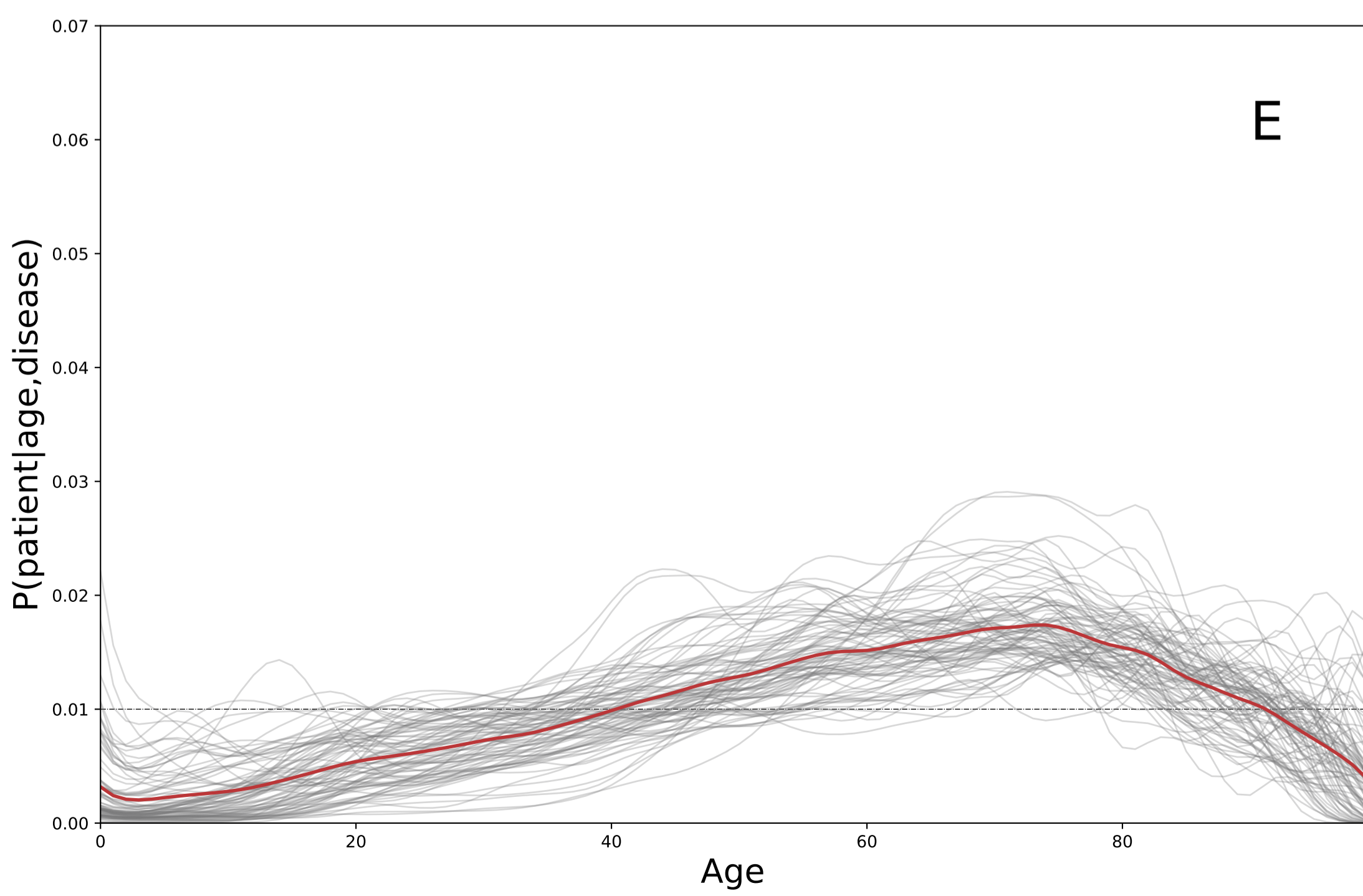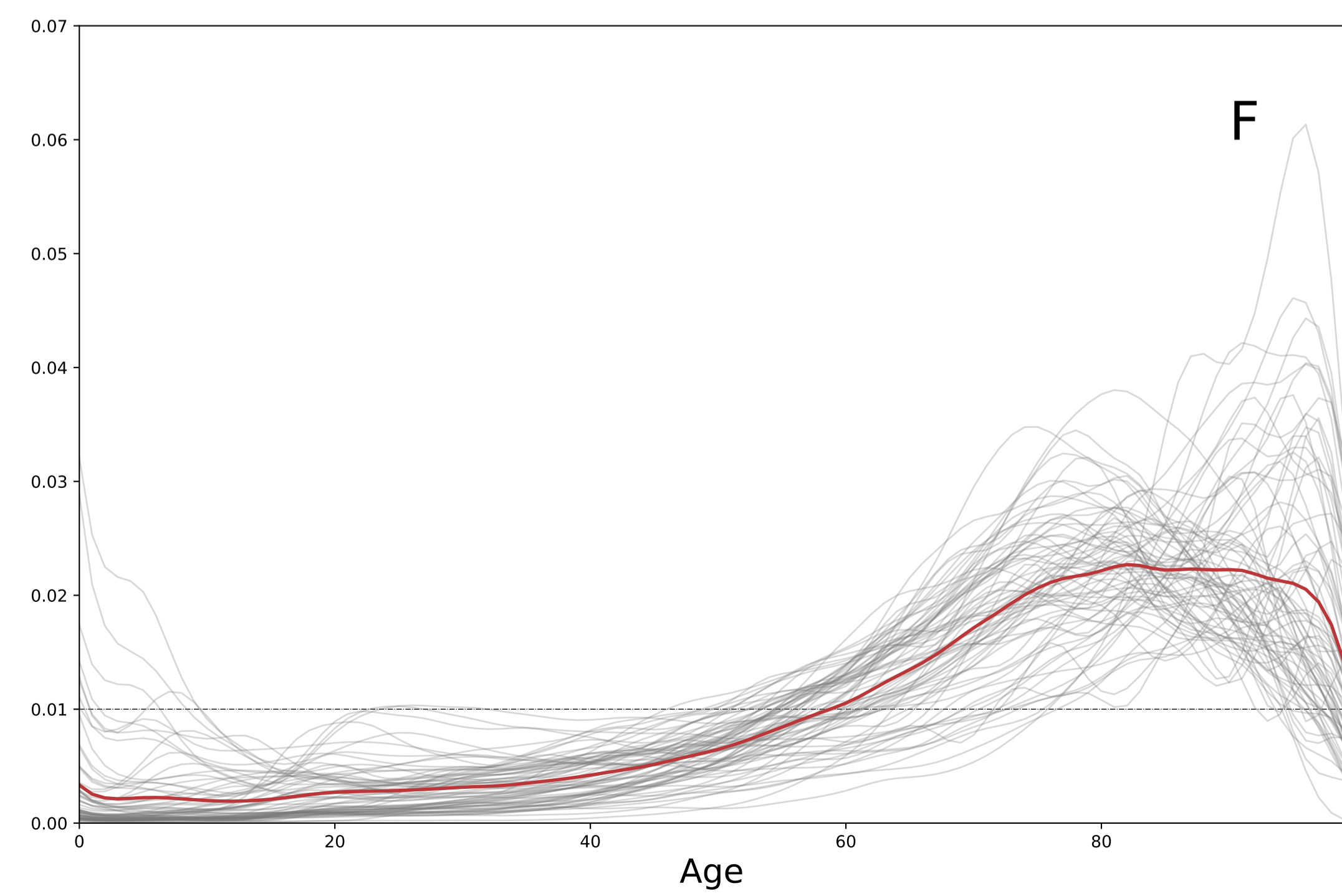

Supplement: S1 Fig — Kernel density estimation for a sample of disease codes from each cluster. Lines in gray represent probability distribution of P(age|patients ∈ c) and lines in red are the cluster averages for illustration. The clusters of ICD-10 codes given by the HAC are labeled from A to F. (PDF) [file pcbi.1006115.s002.pdf]
